# Bilateral Sequential Abducens Nerve Palsy After Pfizer-BioNTech COVID-19 Vaccine (BNT162b2): A Case Report and Literature Review

**DOI:** 10.7759/cureus.51682

**Published:** 2024-01-05

**Authors:** Malak F Albattah, Kefah Al-Hayk, Mutasim Albattah, Mohammad A Alshrouf

**Affiliations:** 1 Neurology, Jordan University of Science and Technology, Irbid, JOR; 2 Faculty of Medicine, Jordan Ministry of Health, Amman, JOR; 3 General Medicine, The University of Jordan, Amman, JOR

**Keywords:** ophthalmoplegia, sixth nerve palsy, abducens nerve palsy, coronavirus, covid-19, vaccination

## Abstract

This case report details the occurrence of bilateral sequential abducens nerve palsy in a previously healthy 42-year-old woman two days after receiving her first dose of the Pfizer-BioNTech COVID-19 vaccine (BNT162b2). Despite the widespread global administration of COVID-19 vaccines, instances of abducens palsy following vaccination are limited in the available literature. Considering the temporal association between vaccination and symptom onset, the absence of underlying medical conditions predisposing to such neurological manifestations, normal brain imaging results, the occurrence of other cranial palsies post-vaccination, and analogous occurrences after different vaccinations, we propose a plausible connection between the patient’s abducens palsy and the COVID-19 vaccination. Our findings contribute to the growing body of evidence regarding the side effects and safety profile of COVID-19 vaccines. Importantly, the resolution of symptoms with conservative management and the uneventful administration of the second vaccine dose suggest that the observed abducens palsy may be a transient and isolated reaction.

## Introduction

Vaccines against SARS-CoV-2 needed to be developed quickly due to the rapid global spread of the virus. Therefore, several vaccinations were created and approved for use less than a year after the virus was discovered [1]. Accordingly, the Centers for Disease Control and Prevention decided to utilize a real-time vaccine adverse event reporting system (VAERS) called V-Safe to monitor any potential vaccine side effects [2].

Cranial nerve palsies are known to occur after various vaccinations, including the most common trivalent inactivated influenza vaccine, human papillomavirus quadrivalent, inactivated influenza A (H1N1), and zoster vaccine [3]. Moreover, isolated abducens nerve injury was the most common injury reported to the US Vaccine Adverse Event Reporting System (VAERS) [3]. Furthermore, viral infections were associated with abducens nerve injury, including COVID-19 and other viral infections [4].

Abducens palsy is the most common ocular cranial nerve palsy to occur in isolation, with an annual incidence of 11.3 per 100,000 [5]. A 15-year retrospective study identified the most common causes of abducens palsy to be undetermined (26%), hypertension alone (19%), coexistent hypertension and diabetes (12%), trauma (12%), multiple sclerosis (7%), neoplasm (5%), diabetes alone (4%), cerebrovascular accident (4%), post-neurosurgery (3%), aneurysm (2%), and others (8%) [6]. A few cases of abducens nerve palsy were reported after COVID-19 vaccination [7-15]; however, bilateral abducens palsy was not previously reported in the literature. In this article, we describe a case of bilateral sequential abducens nerve palsy after the first dose of the Pfizer-BioNTech COVID-19 vaccine (BNT162b2) and a review of the previously published cases.

## Case presentation

A previously healthy 42-year-old female patient presented with bilateral sequential abducens nerve palsy two days after receiving her first dose of the Pfizer-BioNTech COVID-19 vaccine (BNT162b2). A day after she received the first dose of the vaccine, she awoke with painless binocular horizontal diplopia and some limitation to the lateral movement (abduction) of her left eye, as described by her husband, which lasted for four to six hours and improved spontaneously. The following day, she awoke with painless binocular horizontal diplopia, worse in the far gaze and the right lateral gaze, but this time she had a limitation to the lateral movement (abduction) of her right eye; this limitation did not improve or worsen with time. Her vaccination was otherwise only complicated by pain at the injection site; she had no fever, chills, fatigue, symptoms of other cranial neuropathies (anosmia, blurred vision, ptosis, facial droop), other focal neurological complaints, or symptoms of increased intracranial pressure (headache, vomiting, tinnitus). She had never experienced similar symptoms before and was not previously infected with COVID-19. The patient did not have any symptoms of COVID-19 infection and had no contact with COVID-19; thus, she was not tested for COVID-19 infection. The patient did not report any history of fluctuating weakness, ptosis, diplopia, or diurnal variation.

**Figure 1:**
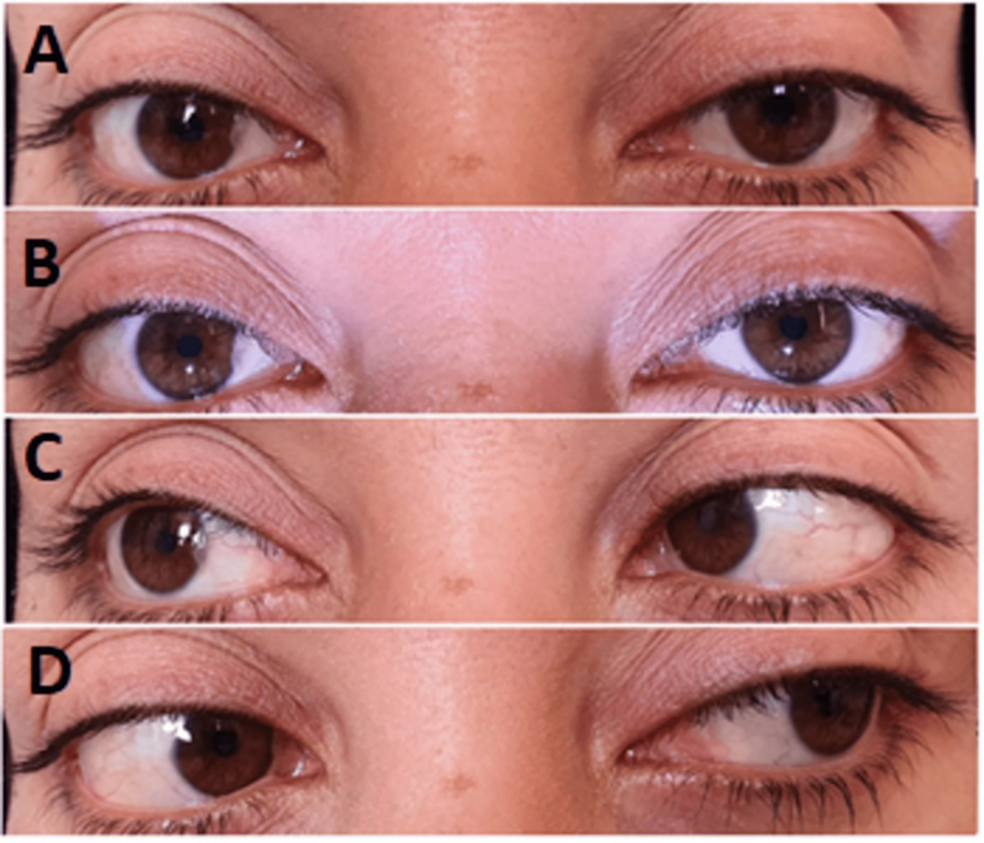
Ocular motility examination demonstrating slight right esotropia on primary gaze (A, B), limitation to the abduction of the right eye (C), and a minimal limitation to the abduction of the left eye (D).

On examination, she had right esotropia on primary gaze, limitation to the abduction of the right eye, and minimal limitation to the abduction of the left eye (Figure 1). A comparison with her old photographs revealed no ptosis. Ophthalmic examination, including slit-lamp and fundus examination, was normal. She underwent a confrontation visual field examination which was normal. Neurological examination was otherwise unremarkable; there was no evidence of other cranial neuropathies, ataxia, or hyporeflexia. The patient did not have any vascular risk factors except for slight obesity; her body mass index was 31.24 kg/m^2^ (weight 78 kg, height 158 cm), random finger stick blood glucometer (ACCU-CHEK®) was in the range 114-127, hemoglobin A1c was 5.1%, low-density lipoprotein was 1.54 mmol/L, and cholesterol was 4.0 mmol/L. Other lab workups included a complete blood count (white cell count 6.5 10^3^/mm^3^, neutrophils 62.8%, lymphocytes 29.8%, hemoglobin 13.5 g/dL, platelets 253), erythrocyte sedimentation rate was not available in the hospital at the time, C-reactive protein was negative, thyroid stimulating hormone was 1.5 mIU/L, and antinuclear antibody and rheumatoid factor were both negative. Kidney function tests, liver function tests, calcium, magnesium, and phosphorus were all within normal limits. Table 1 summarizes the laboratory results and the reference values.

**Table 1:** Patient’s lab results along with their reference values.

Test name	Unit	Result	Reference range
White cell count	10^3^/mm^3^	6.50	3.50–11.00
Red blood corpuscles	10^6^/mm^3^	4.39	3.80–5.50
Hemoglobin	g/dL	13.50	11.00–16.00
Hematocrit	%	38.40	34.00–47.00
Mean cell volume	μm^3^	87.50	78.00–100.00
Mean cell hemoglobin	pg/cell	30.70	27.00–35.00
Mean cell hemoglobin concentration	g/dL	35.10	31.00–37.00
Red cell width	%	13.10	11.50–17.00
Platelets	10^3^/mm^3^	253.0	150.0–400.0
Mean platelet volume	μm^3^	8.60	6.00–10.00
Lymphocytes	%	29.80	20.00–45.00
Monocytes	%	5.90	2.00–6.00
Neutrophils	%	62.80	60.00–75.00
Eosinophils	%	1.00	1.00–3.00
Basophils	%	0.50	0.00–1.00
Sodium	mmol/L	139.00	135.00–145.00
Potassium	mmol/L	4.42	3.30–5.10
Urea	mmol/L	2.60	2.14–7.14
Creatinine	μmol/L	51.00	53.00–97.00
Calcium	mmol/L	2.35	2.15–2.50
Magnesium	mmol/L	0.76	0.66–1.07
Phosphorus	mmol/L	0.82	0.81–1.45
Uric acid	μmol/L	182.00	142.00–339.20
Total bilirubin	μmol/L	9.30	5.00–21.00
Direct bilirubin	μmol/L	3.80	0–5
Alkaline phosphatase (total)	U/L	73	35–104
Alanine transaminase	U/L	24.9	0.0–33.0
Aspartate aminotransferase	U/L	22.1	0.00–32.00
Gamma-glutamyl transferase	U/L	15.00	5.00–36.00
Hemoglobin A1C	%	5.1	<5.7
Thyroid-stimulating hormone	mU/L	1.5	0.5–5.0
High-density lipoprotein cholesterol	mmol/L	1.36	0.9–1.2
Low-density lipoprotein cholesterol	mmol/L	1.54	<3.4
Cholesterol	mmol/L	4.01	<5.2
Triglycerides	mmol/L	2.44	<1.7
C-reactive protein	mg/L	Negative	-
Anti-nuclear antibody	Titer	Negative	-
Anti-DNA	Titer	Negative	-
Rheumatoid factor	IU/mL	Negative	-

Magnetic resonance imaging (MRI) with enhancement and magnetic resonance (MR) angiography of the brain were normal. She declined an inpatient lumbar puncture. She was diagnosed with abducens palsy, with a possible association with COVID-19 vaccination given the temporal relationship and unremarkable workup.

She was managed conservatively with a wait-and-see approach; steroids were not given due to a lack of evidence of such practice in the treatment of non-cranial nerve VII palsies. The abducens palsy completely resolved within two months. On follow-up, the patient had a normal primary eye position. She also received the second dose of the Pfizer-BioNTech COVID-19 vaccine three months after the first dose with no complications.

## Discussion

To date, billions of people worldwide have received at least one dose of the COVID-19 vaccine. There are, however, only nine reported cases of the COVID-19 vaccine causing isolated abducens palsy [7-15]. However, none of the previous cases reported bilateral abducens nerve palsy. On the other hand, there are more reported cases of COVID-19 infection causing abducens palsy, including cases of isolated abducens palsy [4], one case with polyneuritis cranialis [16], two cases with Miller Fisher syndrome [16,17], one case with Wernicke’s encephalopathy in a patient with bulimia [18], one case with mammillary body and hypothalamic involvement [18], and one case with reversible leukoencephalopathy [19].

The usual presentation has been reported to be within a week of receiving the COVID-19 vaccine, with three expectations: Ginés-Gallego et al. reported abducens palsy 15 days after COVID-19 Pfizer-BioNTech vaccination [15]; Khalili et al. reported a case of isolated abducens palsy 10 days after Sinopharm vaccine; and they mentioned an unpublished case of abducens palsy that occurred 14 days after Bharat Biotech BBV152 (Covaxin) COVID-19 vaccine [12]. They attributed this delay to the time of vaccine-triggered inflammation, immune complex deposition, and microvascular thrombosis [12]. In most of the previously published case reports, symptoms resolved within 5-12 weeks with only conservative treatment. Table 2 summarizes the treatment modalities and outcomes of the published cases of isolated abducens palsy after COVID-19 vaccination.

**Table 2:** Summary of the treatment modalities and outcomes of the published cases of isolated abducens palsy after COVID-19 vaccination.

Article	Age	Type of vaccine	Side	Symptoms appear after the vaccine	Treatment	Reported outcome
Reyes-Capo et al., 2021 [7]	59	Pfizer- BioNTech	Right Eye	2 days	Not reported	No improvement
Pereira and Haslett, 2021 [8]	65	AstraZeneca	Right eye	3 days	Not reported	Resolved within 3 months
Pawar et al., 2021 [9]	23	Covishield	Left eye	7 days	Not reported	Improved to near-normal
Karam et al., 2022 [10]	46	Pfizer- BioNTech	Left eye	4 days	No treatment	Resolved within 5 weeks
Veisi et al., 2022 [11]	55	Sinopharm	Right eye	7 days	No treatment	Resolved within 2 months
Khalili et al., 2022 [ 12]	63	Sinopharm	Left eye	10 days	Not reported	Not reported
Kakil and Kosker, 2022 [13]	57	Not reported	Right eye	2 days	Aspirin	Resolved within 3 weeks
Basnet et al., 2022 [14]	72	AstraZeneca	Right eye	3 days	Alternate patching of the eye	Improving
Ginés-Gallego et al., 2022 [15]	75	Pfizer- BioNTech	Left eye	15 days	Botulinum toxin A (5 U) injection	Improving

Cranial nerve palsies were reported after several types of vaccines, and, recently, they were reported after the COVID-19 vaccination [3,4]. Except for facial palsy, abducens palsy was the most common isolated motor cranial nerve palsy and the most cranial nerve palsy reported to the US VAERS, followed by oculomotor and trochlear nerve palsies. Serious cases were more likely to be reported than non-serious ones, and, accordingly, it seemed plausible that there could be substantial underreporting of cases. Although rare, the effects of abducens palsy can be distressing to the patient, causing impairment of far vision and the ability to perform instrumental activities of daily living such as driving.

The pathophysiology of abducens palsy following vaccination can be elucidated from the more extensively studied Bell’s palsy following COVID-19 vaccination, which is hypothesized to be via either mimicry of host molecules, bystander activation of dormant autoreactive T cells, or induction of innate immune activation and production of interferon proteins by a combined effect of mRNA and lipids [20]. According to previous research, there are three primary postulated processes underlying the development of CN6 palsy in connection to COVID-19 [4]. These mechanisms include inflammation or autoimmune injury caused by abnormal and excessive inflammation in COVID-19, potentially due to a cross-reaction between the SARS-CoV-2 spike protein and myelin basic protein. The second mechanism involves coagulopathy, which is caused by vascular endothelial injury. This injury triggers a condition of procoagulable state, leading to cerebrovascular injury. Additionally, the virus may directly damage the central nervous system by invading it, potentially affecting the six cranial nerves.

## Conclusions

The manifestation of bilateral sequential abducens palsy two days following the administration of the BNT162b2 vaccine presents a noteworthy clinical scenario and, interestingly, the absence of an overactive immune response such as fever. After a comprehensive evaluation of the case, it was thought to be related to her vaccination, taking into account the temporal relationship between the vaccination and the onset of symptoms, the absence of medical illnesses known to predispose to such a condition, the unremarkable brain imaging, the occurrence of other cranial palsies after the same vaccine, and the occurrence of similar palsies after other vaccinations.

It is crucial to emphasize the broader significance of the present case within the emerging landscape of post-vaccination complications. Significantly, there have been reports of similar cranial palsies occurring after the administration of the BNT162b2 vaccine, as well as cases of similar neurological problems linked to other vaccines. This highlights the need for a comprehensive investigation into possible causative linkages.
